# Mural Cells Initiate Endothelial-to-Mesenchymal Transition in Adjacent Endothelial Cells in Extracranial AVMs

**DOI:** 10.3390/cells13242122

**Published:** 2024-12-21

**Authors:** Syed J. Mehdi, Haihong Zhang, Ravi W. Sun, Gresham T. Richter, Graham M. Strub

**Affiliations:** 1 Arkansas Children’s Research Institute (ACRI), Little Rock, AR 72202, USA; sjmehdi@uams.edu (S.J.M.);; 2 Department of Otolaryngology-Head and Neck Surgery, College of Medicine, University of Arkansas for Medical Sciences, Little Rock, AR 72202, USA

**Keywords:** extracranial arteriovenous malformation, mural cells, EndMT, endothelial cells, CD31, vascular instability, vascular anomaly

## Abstract

Extracranial arteriovenous malformations (eAVMs) are complex vascular lesions characterized by anomalous arteriovenous connections, vascular instability, and disruptions in endothelial cell (EC)-to-mural cell (MC) interactions. This study sought to determine whether eAVM-MCs could induce endothelial-to-mesenchymal transition (EndMT), a process known to disrupt vascular integrity, in the eAVM microenvironment. eAVM and paired control tissues were analyzed using RT-PCR for EC (*CD31*, *CD34*, and *CDH5*) and EndMT-specific markers (*SNAI1*, *SNAI2*, *ACTA2/α-SMA*, *N-cadherin*/*CDH2, VIM)*. Immunohistochemistry (IHC) was also performed to analyze MC- (PDGFR-β and α-SMA), EC (CD31, CD34, and CDH5), and EndMT-specific markers (CDH2 and SNAI1) in sequential paraffin-embedded sections of eAVM patient biopsies and in adjacent normal tissue biopsies from the same patients. Furthermore, eAVM-MCs and MCs from normal paired tissues (NMCs) were then isolated from fresh human surgical samples using flow cytometry and co-cultured with normal human umbilical vein vascular endothelial cells (HUVECs), followed by analysis of CD31 by immunofluorescence. RT-PCR analysis did not show a significant difference in the expression of EC markers between eAVM tissues and controls, whereas expression of EndMT-specific markers was upregulated in eAVM tissues compared to controls. IHC of eAVMs and paired control tissues demonstrated regions of significant perivascular eAVM-MC expansion (PDGFR-β+, and α-SMA+) surrounding dilated, morphologically abnormal vessels. These regions contained endothelium undergoing EndMT as evidenced by loss of CD31, CD34, and CDH5 expression and upregulation of the EndMT-associated genes CDH2 and SNAI1. Isolated eAVM-MCs induced loss of CD31 in HUVECs when grown in co-culture, while NMCs did not. This study suggests that the eAVM endothelium surrounded by expanded eAVM-MCs undergoes EndMT, potentially leading to the formation of dilated and fragile vessels, and implicates the eAVM-MCs in EndMT initiation and eAVM pathology.

## 1. Introduction

Extracranial arteriovenous malformations (eAVMs) are congenital high-flow vascular malformations containing a complex network of aberrantly connected arteries and veins without normal intervening capillary vasculature [1,2,3]. eAVMs occur most commonly in the head and neck and are the most morbid vascular anomalies due to their aggressive growth and tendency to hemorrhage [4]. eAVMs are present at birth and progress during adolescence and adulthood, often requiring multiple interventions throughout the patient’s life [1,5,6]. eAVMs contain a mixture of dilated, pathologically friable blood vessels (BVs) that infiltrate surrounding normal tissue and gradually enlarge, often leading to pain, ulceration, disfigurement, tissue destruction, obstruction of vital structures, and even congestive heart failure [7,8,9]. The destructive nature of eAVMs and their tendency to hemorrhage or recur after traditional interventions highlights the need to develop interventions that target the molecular processes that result in eAVM vessel pathology, which is poorly understood.

eAVM formation parallels normal BV formation in that it involves multiple cell types, such as endothelial cells (ECs) and vascular mural cells (MCs (pericytes (PCs) and vascular smooth muscle cells (VSMCs)) that control both the development and pathologic disease states of BVs [10]. Development of the vascular system initiates with the formation of EC-line tubes, either from new BVs through vasculogenesis or by sprouting of new vessels from pre-existing parental vessels through angiogenesis [10,11]. After these EC tube networks are formed, MC recruitment occurs along the tube abluminal surface. In angiogenesis, sprouting ECs secrete platelet-derived growth factor (PDGF) to recruit platelet-derived growth factor receptor-beta (PDGFR-β) positive MCs, which then interact with ECs and stabilize the newly formed BVs [12]. This represents a critical event controlling further tube remodeling, maturation, and stabilization [13]. Such vessel stability is attained through EC–MC interactions and concomitant extracellular matrix (ECM) remodeling, including deposition and cross-linking of ECM components [14,15,16]. Any aberrations during this process can lead to thin and friable vessels that tend to bleed with minimal disruption, which is the typical phenotype of eAVMs [17,18]. MC dysfunction has been implicated in other vascular diseases including brain AVMs (bAVMs) [19,20,21,22,23,24,25], and eAVM-MCs demonstrate alterations in expression of mesenchymal markers such as smooth muscle actin (αSMA) [26]. The possibility that eAVM-MCs are driving eAVM pathology opens a potential new avenue for molecular interventions, which necessitates a deeper mechanistic understanding of the associated molecular, cellular, and functional changes that occur in MCs during eAVM progression.

Endothelial-to-mesenchymal transition (EndMT) is a process in which ECs lose their cell-type-specific characteristics and acquire a mesenchymal and stem-cell-like phenotype [27,28]. EndMT in vascular ECs predictably results in pathology due to the loss of expression of genes necessary for the maintenance of vascular integrity. EndMT contributes to endothelial dysfunction in cardiovascular diseases [29,30,31,32] and occurs in the endothelium of other vascular anomalies such as cerebral cavernous malformations (CCMs) and bAVMs [33,34,35,36], but the contribution, mechanism, and therapeutic potential of targeting EndMT in eAVMs remains unknown.

We sought to determine whether EndMT is occurring in eAVMs and to elucidate the contribution of the perivascular eAVM-MCs to that process. We demonstrate that eAVMs contain a mixture of BVs, which are of either normal caliber or which are grossly dilated. These dilated vessels are lined by ECs that have lost the expression of the mature EC markers CD31 and CD34 and are surrounded by a significantly enlarged perivasculature containing PDGFR-β-positive eAVM-MCs. These CD31- ECs undergo EndMT as evidenced by expression of several EndMT-associated transcription factors and mesenchymal markers. Finally, co-culture of HUVECs with isolated eAVM-MCs, but not wildtype MCs, induced loss of HUVEC CD31 expression. These results suggest that eAVMs contain an abnormally enriched MC layer that induces EndMT in the adjacent endothelium, highlighting EndMT inhibition in mural cells as a novel potential molecular target in both eAVMs and other vascular diseases.

## 2. Materials and Methods

### 2.1. Study Approval

All human subjects signed written informed consent giving permission for tissue collection, use of their biologic specimens, and cellular and genetic analyses, according to Institutional Review Board protocols (University of Arkansas for Medical Sciences Institutional Review Board number 114012).

### 2.2. Primary Human Samples

Extracranial AVM tissues and normal tissue biopsies (harvested from adjacent, unaffected subcutaneous tissue) were collected from patients undergoing surgical resections and deidentified. The cohort consisted of pediatric patients, and the biopsies were collected from head and neck regions. After surgical harvest, tissue specimens were divided and immediately put into 10% neutral formalin for histological staining, flash frozen in liquid nitrogen, and transferred to a − 80 °C freezer for RT-PCR, or collected in normal saline at 4 °C for cell isolation.

### 2.3. Isolation of Mural Cells from Extracranial AVM and Paired Normal Specimens

MCs were isolated from eAVM lesions (*n* = 3) and their paired normal tissues as previously described [37]. Briefly, tissues were dissociated with 0.25% collagenase I (Worthington Biochemical, Lakewood, NJ, USA) in explant medium (DMEM medium (Thermo-Fisher Scientific, Waltham, MA, USA) supplemented with 20% fetal bovine serum (FBS, R&D Systems, Inc., Minneapolis, MN, USA) and 1% antibiotic-antimycotic (Thermo-Fisher Scientific, Waltham, MA, USA) at room temperature for 1 h with agitation. Dissociated cells were filtered through a 100 μm cell strainer (Corning, Glendale, AZ, USA) and then cultured in pericyte medium (Sciencell, Carlsbad, CA, USA) and further passaged to grow sufficient MCs for the co-culture experiment.

### 2.4. Flow Cytometry

We isolated PDGFR-β+ MCs from cells expanded from eAVM lesions (*n* = 3) and their paired normal tissues (*n* = 3). Cells were stained with PE-conjugated PDGFR-β antibody (Table S1) and analyzed with flow cytometry (FACSAria Fusion, BD Biosciences, San Jose, CA, USA). Sorted PDGFR-β+ cells were further cultured and expanded for co-culture experiments.

### 2.5. Cell Culture

Human umbilical vascular endothelial cells (HUVECs) were purchased (PromoCell, Heidelberg, Germany) and cultured in EBM2 medium (Lonza, Morristown, NJ, USA) supplemented with 20% fetal bovine serum (FBS, R&D Systems, Inc., Minneapolis, MN, USA), 1% antibiotic-antimycotic (Thermo-Fisher Scientific, Waltham, MA, USA), and other growth factors. HUVECs were further passaged for co-culture experiments. Two-dimensional co-culture was performed as previously described [38,39]. Briefly, HUVECs and isolated MCs from three different eAVM patients and paired normal tissue were plated in 24-well plates (Corning, Glendale, AZ, USA) and cultured in EBM2 and pericyte medium in a 1:1 ratio for 5 days. Indirect co-culture was performed using these cells with 0.4 µm PET insert in between (Millipore, Burlington, MA, USA). After 5 days of culture, cells were fixed for immunofluorescence.

### 2.6. Immunofluorescence Staining

Immunofluorescence staining was performed as described previously [40]. Briefly, cells were fixed with 4% paraformaldehyde for 15 min after co-culture, permeabilized with 0.25% Triton X-100 in PBS for 10 min, and blocked in 5% BSA for 1 h. Cells then were incubated overnight at 4 °C with anti-CD31 and anti-PDGFR-β antibodies (Thermo Scientific, Waltham, MA, USA). The next day, cells were washed with PBS and incubated in the dark for 2 h with a mixture of 2 fluorescent secondary antibodies—FITC-conjugated anti-mouse IgG (Thermo Scientific, Waltham, MA, USA) for detecting CD31 and CD13 and TRITC-conjugated anti-rabbit IgG (Thermo Scientific, Waltham, MA, USA) for detecting PDGFR-β cells. Details of the antibodies used are summarized in Table S1. Cell nuclei were stained with DAPI (Thermo Scientific, Waltham, MA, USA). An Olympus BX43 microscope (Olympus, Tokyo, Japan) was used to obtain images at 20x magnification with an Infinity 3S digital camera (Teledyne Lumenera, Ottawa, ON, Canada). Images were taken using Infinity Analyze v7.1 software (Teledyne Lumenera, Ottawa, ON, Canada) and further processed to 600 dpi using Photoshop CS6 (64 bit) (Adobe, San Jose, CA, USA). The fluorescence intensity was quantified using ImageJ software v1.54k.

### 2.7. Total RNA Isolation and Real-Time PCR

About 30 mg of tissue was used for the purification of total RNA by RNeasy Plus Universal Mini Kit (Qiagen, Germantown, MD, USA). RNA concentration was measured using the NanoDrop ND-1000 (Thermo Fisher Scientific, Waltham, MA, USA). All total RNA samples were then converted into cDNA at the same time by High-Capacity cDNA Reverse Transcription Kit (Thermo Fisher Scientific, Waltham, MA, USA). The real-time PCR amplification was performed on QuantStudio 6 Flex Real-Time PCR System (Thermo Fisher Scientific, Waltham, MA, USA). The thermal cycling conditions were 1 cycle of 95 °C 10 min, followed by 40 cycles of 95 °C for 15 s and 60 °C for 1 min. The total reaction volume of 10 µL contained 5 ng of cDNA template, 5 µL of 2× TaqMan Universal Master Mix II (No UNG) (Thermo Fisher Scientific, Waltham, MA, USA), and 0.5 µL of 20× primer/probe assay (Thermo Fisher Scientific, Waltham, MA, USA). All primers used for the mRNA assay are shown in Table S2. GAPDH (Hs03929097_g1) was used as the endogenous control. The relative standard curve was used for relative quantitation. The amplification amount of mRNA was normalized against that of GAPDH.

### 2.8. Immunohistochemistry

Immunohistochemistry (IHC) staining was performed on paraffin sections as described previously [39,41]. Briefly, after performing heat-mediated antigen retrieval with Tris/EDTA buffer (pH 9.0) for 30 min, slides were blocked with normal blocking serum for 20 min, followed by incubation with the desired antibody for 2 h after serial washing for 5 min. All the antibodies used are shown in Table S1. Assays were completed with a Vectastain elite ABC peroxidase (HRP) kit (Vector Laboratories, Burlington, ON, Canada) and counterstaining with hematoxylin. Images were collected using an Olympus BX43 microscope (Olympus, Tokyo, Japan) at 20× and 40× magnification with an Infinity 3S digital camera (Teledyne Lumenera, Ottawa, ON, Canada) in bright-field mode. Images were taken using Infinity Analyze 7 software (Teledyne Lumenera, Ottawa, ON, Canada) and further processed to 600 dpi using Photoshop CS6 (64 bit) (Adobe, San Jose, CA, USA).

### 2.9. Statistical Analysis

All values are expressed as mean ± standard error of the mean unless indicated otherwise. A paired two-tailed *t*-test was used to analyze the quantitative RT-PCR data for mRNA expression using GraphPad Prism 9.4.0 (GraphPad Software, San Diego, CA, USA). Differences were considered statistically significant at *p* < 0.05.

## 3. Results

### 3.1. CD31-eAVM-ECs Are Surrounded by an Expanded Perivasculature of MCs

**Figure 1:**
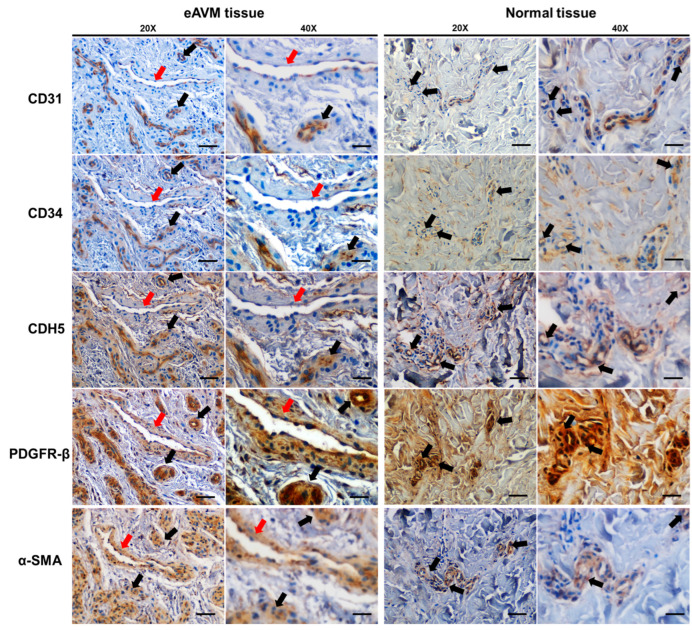
Analysis of mature EC markers and MC markers in consecutive sections of eAVM biopsies and normal adjacent tissue biopsies. Adjacent paraffin sections of eAVM tissue and normal tissue harvested from a nearby unaffected region were stained for the mature EC markers CD31 (first row), CD34 (second row), and CDH5 (third row) and MC markers PDGFR-β+ (fourth row) and α-SMA (fifth row). Examples of normal caliber CD31+/CD34+ EC-lined vessels are indicated by black arrows, and dilated CD31-/CD34- vessels are indicated with red arrows. CD31+/CD34+/CDH5+ vessels are surrounded by a thin layer of PDGFR-β+ and α-SMA+ MCs (black arrows), while dilated CD31-/CD34-/CDH5- vessels are surrounded by an expanded PDGFR-β+ and α-SMA+ (red arrows) MC perivasculature. Magnification = 20× and 40×; bar = 200 μm and 100 μm.

EC–MC communication is essential for vascular development, and their defective association results in multiple human diseases [18,42,43,44,45,46]. To illustrate the EC and MC composition of eAVMs compared to adjacent normal tissues, we first examined the expression of EC markers CD31, CD34, and VE-Cadherin (CDH5) [47,48,49] and the MC markers PDGFR-β [50] and α-SMA [51] in sequential paraffin-embedded sections of eAVM patient biopsies and in adjacent normal tissue biopsies from the same patients using IHC. eAVM sections contained a mixture of both large, dilated “abnormal” appearing BVs and regions of “normal” appearing, smaller BVs, similar to the BVs observed in the normal tissue biopsies. The expression of EC markers was detected in all samples by IHC but was confined to the vessels of normal caliber (Figure 1). The endothelium of the morphologically abnormal vessels did not express CD31, CD34, or CDH5 (Figure 1). RT-PCR analysis did not demonstrate a significant difference in the expression of EC markers between eAVM tissues and controls (Figure S1). Staining for the MC marker PDGFR-β demonstrated that all CD31+CD34+ endothelium-lined BVs (found in both eAVM and control tissue) were surrounded by a thin perivascular layer. In contrast, CD31-CD34-endothelium-lined BVs (found only in eAVM samples) were surrounded by a dramatically expanded perivascular layer (Figure 1). IHC staining for α-SMA showed a similar staining pattern to that observed for PDGFR-β, confirming that all CD31+CD34+ endothelium-lined BVs (found in both eAVM and control tissue) were surrounded by a thin perivascular layer, while CD31-CD34-endothelium-lined BVs (found only in eAVM samples) were surrounded by a dramatically expanded perivascular layer (Figure 1). This suggests eAVMs contain a population of morphologically abnormal BVs lined by ECs that have lost expression of CD31, CD34, and CDH5, a known hallmark of EndMT, and are surrounded by an expanded eAVM-MC perivasculature.

### 3.2. CD31- eAVM-ECs Undergo EndMT

**Figure 2:**
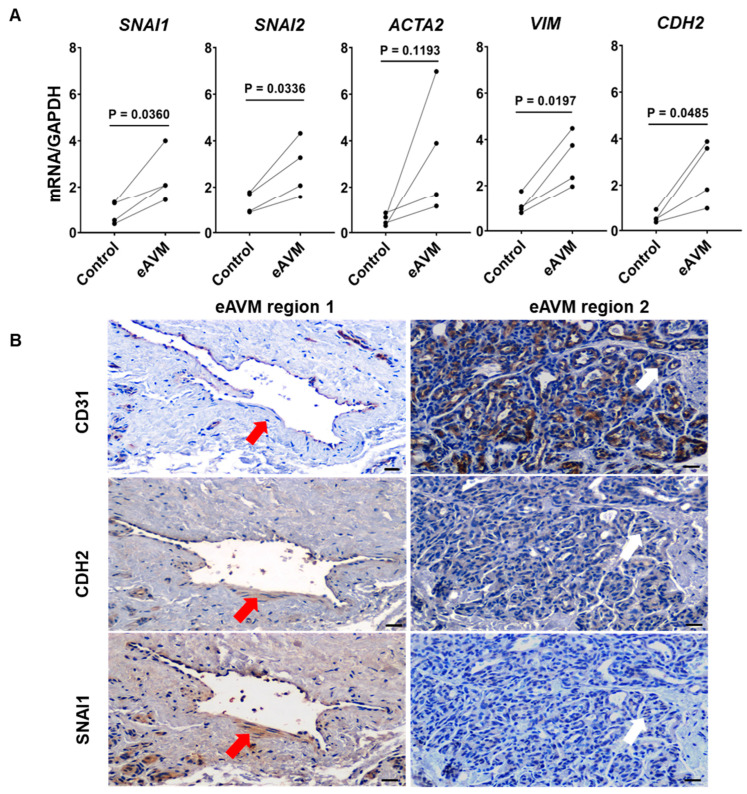
(**A**). EndMT-associated markers are elevated in eAVM tissues compared to paired control tissues. Expression levels of EndMT-associated transcription factors *SNAI1* and *SNAI2* and mesenchymal markers *ACTA2*, *VIM*, and *CDH2* were analyzed by RT-PCR. Paired two-tailed *t*-tests were performed to determine statistical significance (*p* < 0.05 was considered significant). (**B**). Expression of EndMT-specific N-cadherin (CDH2) and SNAI1 occurred in the endothelium of dilated CD31- eAVM BVs but not in regions of CD31 expression. Adjacent paraffin consecutive sections of eAVM tissue from three patients were stained with anti-CD31, anti-CDH2, and anti-SNAI1. Red arrows indicate ECs from the same region of eAVM containing a dilated vessel with expanded perivasculature. White arrows indicate ECs from an adjacent region from the same eAVM specimen containing a high density of CD31+ ECs. Magnification = 20×; bar = 200 μm.

EndMT is a complex process characterized not only by the loss of endothelial surface markers but also the upregulation of several EndMT-associated transcription factors, mesenchymal proteins, and extracellular matrix (ECM) proteins. To determine whether the loss of CD31 expression in eAVM-ECs is due to EndMT, we first compared the expression of the EndMT-specific transcription factors *SNAI1* and *SNAI2* [52] between eAVM and normal adjacent tissues using RT-PCR (Figure 2A). We observed significantly higher expression of *SNAI1* (*p* < 0.03) and *SNAI2* (*p* < 0.03) in eAVM tissues compared to adjacent tissue controls. In addition, the mesenchymal genes smooth muscle alpha-2 actin (*ACTA2/α-SMA*) [51], vimentin (*VIM*) [53], and N-cadherin (*CDH2*) [54,55] were all upregulated in eAVM tissues compared to adjacent tissue controls, although *ACTA2* did not reach statistical significance.

To determine whether EndMT was occurring in eAVM-ECs, we performed IHC on eAVM tissues for the EndMT-specific markers such as SNAI1, CDH2, and endothelial CD31 and found that SNAI1 and CDH2 was significantly upregulated in CD31- eAVM-ECs compared to CD31+ ECs (Figure 2B). Again, CD31 expression was maintained in the ECs of adjacent normal appearing BVs but lost in the eAVM-ECs that upregulated SNAI1 and CDH2. These findings suggest that the CD31- eAVM-ECs surrounded by expanded MC perivasculature are undergoing EndMT.

### 3.3. eAVM-MCs Can Induce EndMT in Adjacent ECs

**Figure 3:**
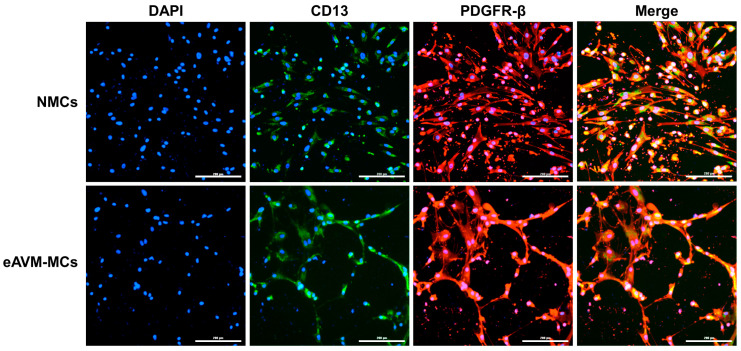
Characterization of mural cell-specific expression of CD13 (green) and PDGFR-β (red) in NMCs (**top row**) and eAVM-MCs (**bottom row**) using immunofluorescence in samples isolated from the same patient. Magnification = 20×; bar = 200 μm.

To determine whether eAVM-MCs could induce EndMT in ECs, we first isolated eAVM-MCs and MCs from normal adjacent tissue biopsies (NMCs), using PDGFRβ as an MC marker from three patients by flow cytometry (Figure S2) and confirmed their identities using immunofluorescence for the MC markers CD13 and PDGFR-β (Figure 3).

**Figure 4:**
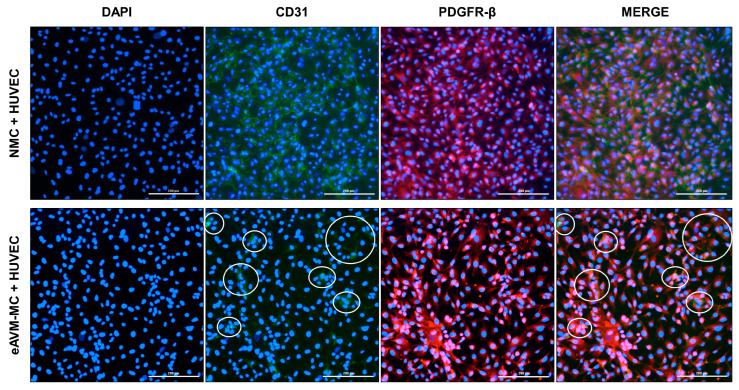
Loss of CD31 (green) expression in HUVECs co-cultured with eAVM-MCs (PDGFR-β red). Representative image shows that CD31 expression was not affected in HUVECs when co-cultured with MCs isolated from normal tissue (NMCs, **top row**), whereas CD31 expression was reduced in HUVECs when co-cultured with eAVM-MCs (**bottom row**). White circles highlight regions of CD31 expression. Magnification = 20×; bar = 200 μm.

We also confirmed the expression of CD31 and PDGFR-β in HUVECs and isolated MCs, respectively, prior to co-culture (Figure S3). To determine whether eAVM-MCs or normal MCs isolated from adjacent tissue biopsies could induce loss of endothelial expression of CD31, we co-cultured isolated MCs with HUVECs and analyzed the expression of CD31 using immunofluorescence (Figure 4). Co-culture of HUVECs with NMCs did not affect the endothelial expression of CD31 (Figure 4, top row), while co-culture with eAVM-MCs resulted in a dramatic reduction of CD31 expression (Figure 4, bottom row). The fluorescence intensity of CD31 in HUVECs also showed a significant decrease in its expression when co-cultured with eAVM-MCs compared to NMCs (Figure S4).

To determine whether this induction of endothelial CD31 expression was mediated by soluble factors released by eAVM-MCs, an indirect co-culture experiment was performed with eAVM-MCs and HUVECs grown on opposite sides of a 0.4 μm permeable membrane (Figure S5). In this indirect co-culture experiment, we did not observe any changes in endothelial CD31 expression in HUVECs cultured with either eAVM-MCs or NMCs, suggesting that the loss of endothelial CD31 expression induced by eAVM-MCs is dependent on EC–MC contact.

## 4. Discussion

EC–MC interaction is critical for normal BV development, and its disruption contributes to numerous disorders such as blood–brain barrier dysfunction [45], diabetic retinopathy [46], and tumor angiogenesis [56]. To understand the EC–MC interaction in the pathogenesis of eAVMs, we collected eAVM tissue specimens along with adjacent unaffected subcutaneous tissue, which appears unaffected by eAVM, as a control from the same patient. The decision to use adjacent unaffected tissue was made to minimize inter-individual variability and better control for genetic and environmental factors, ensuring that the comparison between diseased tissue and control tissue came from the same patient. This approach helps reduce the confounding factors that could arise from using tissue from unrelated patients. Additionally, using tissue from patients with different underlying conditions could introduce genetic and physiological differences, making direct comparisons difficult. Furthermore, obtaining age- and region-matched controls from patients without eAVMs is challenging due to difficulties in acquiring appropriate tissue from non-diseased patients undergoing similar surgical procedures. Given the limited cohort size, we recognize that this represents a key limitation of the present study. Since eAVM is a rare vascular disorder, it is challenging to harvest both diseased and normal samples from the same patient. Larger studies with more diverse control groups are necessary to substantiate the findings from this work.

While several reports have demonstrated that reduced MC coverage of BVs and expansion of pericytes is associated with bAVMs in rodent models [20,22,23], no reports have demonstrated this in human eAVMs. This study illustrates that eAVM tissues consist of both “normal” appearing BVs lined by CD31+ ECs and “abnormal” dilated BVs lined by CD31- ECs, the latter of which are surrounded by a dramatically expanded MC layer. In addition to occurring in several cancers and inflammatory processes [57,58,59], the loss of endothelial CD31 has been observed in other pathologies of the vascular system including vasculitis [60] and formation of neovessels during atherosclerosis [61,62], suggesting that the CD31- regions of eAVMs are distinct and potentially pathologic. While a previous study demonstrated an increase in CD31 protein in eAVM-ECs [63], these cells were isolated using CD31 immunoprecipitation, which would not isolate the CD31- eAVM-ECs that we have observed in proximity to the expanded MC layer. Our study did observe an increase in *CD31* mRNA in eAVM whole tissue samples (Figure S1) compared to controls, likely due to the presence of a large number of CD31+ “normal” eAVM vessels present in these samples. However, we also observed reduced expression of other EC markers such as *CD34* and *CDH5* in some eAVM tissues (Figure S1). The reduced CDH5 expression in the abnormal dilated BVs of eAVM sections (Figure 1) suggest that these BVs are undergoing the process of EndMT.

Our observation that only the eAVM-ECs adjacent to expanded eAVM-MC regions lose CD31 expression raises the possibility that eAVM-MCs induce pathologic changes in the adjacent endothelium. This is consistent with previous studies demonstrating the ability of MCs to modulate EC function [64,65] and the pathologic effects of CD31 loss on BV integrity [66,67,68,69]. This is the first study to demonstrate that eAVM-MCs, but not MCs, isolated from adjacent normal tissue (NMC), induce loss of CD31 in ECs when grown in co-culture (Figure 4). This induction appears to require EC–MC contact, as indirect co-culture of eAVM-MCs with HUVECs across a permeable membrane did not induce loss of endothelial CD31 (Figure S5).

During EndMT, cells not only lose expression of endothelial cell surface markers but also upregulate several EndMT-related transcription factors and other mesenchymal genes [28,70,71,72,73,74], including *SNAI1*, *SNAI2*, *ACTA2*, *VIM*, and *CDH2*, all of which were upregulated in our eAVM tissue biopsies (Figure 2). Because the loss of normal EC surface markers precluded the isolation of CD31- eAVM-ECs by flow cytometry, IHC for both CD31 and EndMT markers CHD2 and SNAI1 was used to illustrate the location of CHD2 and SNAI1 expression and EndMT induction (Figure 2). Interestingly, only the regions of eAVMs that contained grossly dilated CD31- BVs surrounded by expanded MC-containing perivasculature expressed CDH2 and SNAI1, while other eAVM regions consisting of smaller CD31+ BVs without thickened MC perivasculature did not. This raises the possibility that the initiation of EndMT in these “normal” appearing CD31+ vessels leads to dilation, MC deposition, and loss of vascular integrity, all of which contribute to the gradual increase in size and bleeding frequency eAVM patients experience.

While the mechanism of EndMT initiation in eAVMs is unknown, low-frequency somatic single nucleotide variants (SNVs) of the RAS gene family, which regulates EndMT, have been identified in both bAVMs and eAVMs [5,75,76,77,78,79,80,81]. Introduction of the KRASG12D SNV into HUVECs triggers EndMT through activation of the TGF-β/BMP-SMAD4 pathway [36], and a previous study from our laboratory demonstrated both upregulation of TGF-β1 and dysregulation of SMAD signaling in the perivasculature of eAVM tissues from 10 patients [82]. A separate study demonstrated that KRASG12D expressing HUVECs could induce EndMT in neighboring wildtype HUVECs through exosomal miRNA-mediated knockdown of PICK1 (protein interacting with PKC) [83]. While that study focused on endothelial-to-endothelial cell communication, the present study demonstrates that eAVM-MCs can induce EndMT in neighboring ECs. This raises the possibility that a small number of SNV-containing eAVM-ECs trigger the propagation of EndMT across a wide distance of genotypically normal tissue, potentially contributing to the rapidity of eAVM development, growth, and associated morbidity. Reversing the initiation of EndMT has been studied in a wide range of diseases, including bAVMs [36], but has not been explored in eAVMs. The lipid-lowering drug lovastatin was found to alleviate EndMT in rat kidneys [84] and attenuated EndMT in KRASG12D-expressing HUVECs by inhibiting TGF-β/BMP-SMAD4 signaling [36]. The repurposing of medications that inhibit EndMT in the treatment of recalcitrant eAVMs warrants further exploration.

## 5. Conclusions

Extracranial arteriovenous malformations (eAVMs) contain a heterogeneous population of both CD31+ and CD31- vascular endothelial cells (eAVM-ECs), the latter of which line grossly dilated vessels and are surrounded by an expanded perivasculature of PDGFRβ+ mural cells (eAVM-MCs). These regions of CD31- eAVM-EC–MC contact undergo endothelial-to-mesenchymal transition (EndMT). Isolated eAVM-MCs induce loss of CD31 in HUVECs in co-culture. This suggests that the initiation of EndMT may trigger the transformation of normal vessels into the friable vessels prone to bleeding that are characteristic of eAVMs and highlights the need to explore agents that reverse EndMT in the treatment of these patients.

## Data Availability

Data are contained within the article and Supplementary Materials.

## Supplementary Materials

The following supporting information can be downloaded at: https://www.mdpi.com/article/10.3390/cells13242122/s1, Figure S1: Expression of CD31 and CD34 in eAVMs tissues: A-B) CD31 and CD34 expression levels were analyzed by RT-PCR, compared between normal tissue and eAVM tissue, and mRNA levels were normalized to those of GAPDH. Paired two-tailed *t*-tests were performed to determine statistical significance (*p* < 0.05 was considered significant). Figure S2: Flow cytometric analysis and live cell sorting of mural cells isolated from eAVM and paired normal specimens. Figure S3: Representative image shows expression of CD31 and PDGFR-β in HUVEC and isolated mural cells before co-culture. Magnification = 20×; bar = 200 μm. Figure S4: Indirect contact of eAVM’s MCs does not affect CD31 expression in HUVECs. Representative image shows that CD31 expression is not reduced in HUVECs when co-cultured with eAVM MCs in an indirect manner. White arrows indicate CD31 expression. White arrows indicate the same cellular location. Magnification = 20× and 40×; bar = 200 μm and 100 μm. Table S1: Antibodies used for immunohistochemistry, flow cytometry, and immunofluorescence. Table S2: Primer/probe for mRNA assay.
