# Supplementary material for: Mural Cells Initiate Endothelial-to-Mesenchymal Transition in Adjacent Endothelial Cells in Extracranial AVMs
Source: Cells. 2024 Dec 21;13(24):2122. doi: 10.3390/cells13242122 (PMC11727354; doi:10.3390/cells13242122)
Supplement: Supplementary file 1 [file cells-13-02122-s001.zip › Revised Supplementary Figures.pdf]

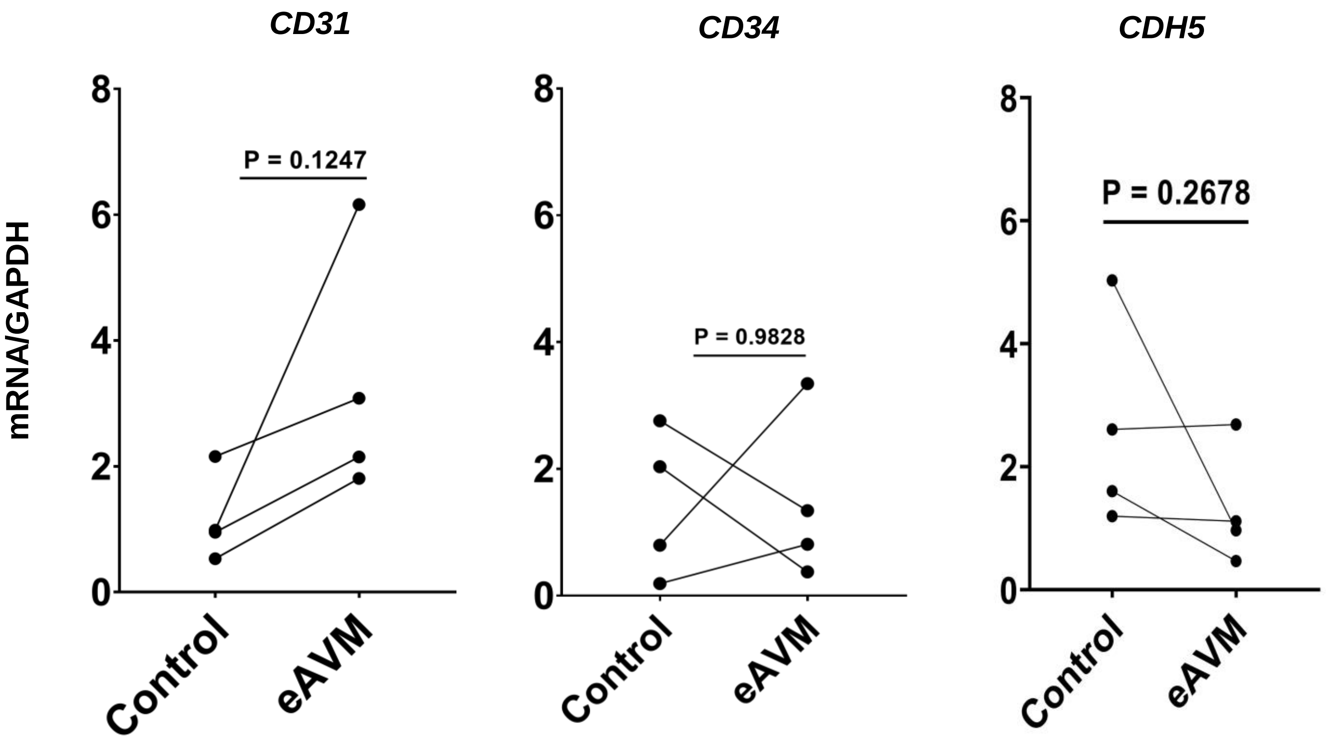

Figure S1. Expression of *CD31, CD34,* and *CDH5* in eAVMs tissues: Expression levels were analyzed by RT-PCR, compared between normal tissue and eAVM tissue, and mRNA levels were normalized to those of *GAPDH*. Paired two-tailed t-tests were performed to determine statistical significance ($P < 0.05$ was considered significant).

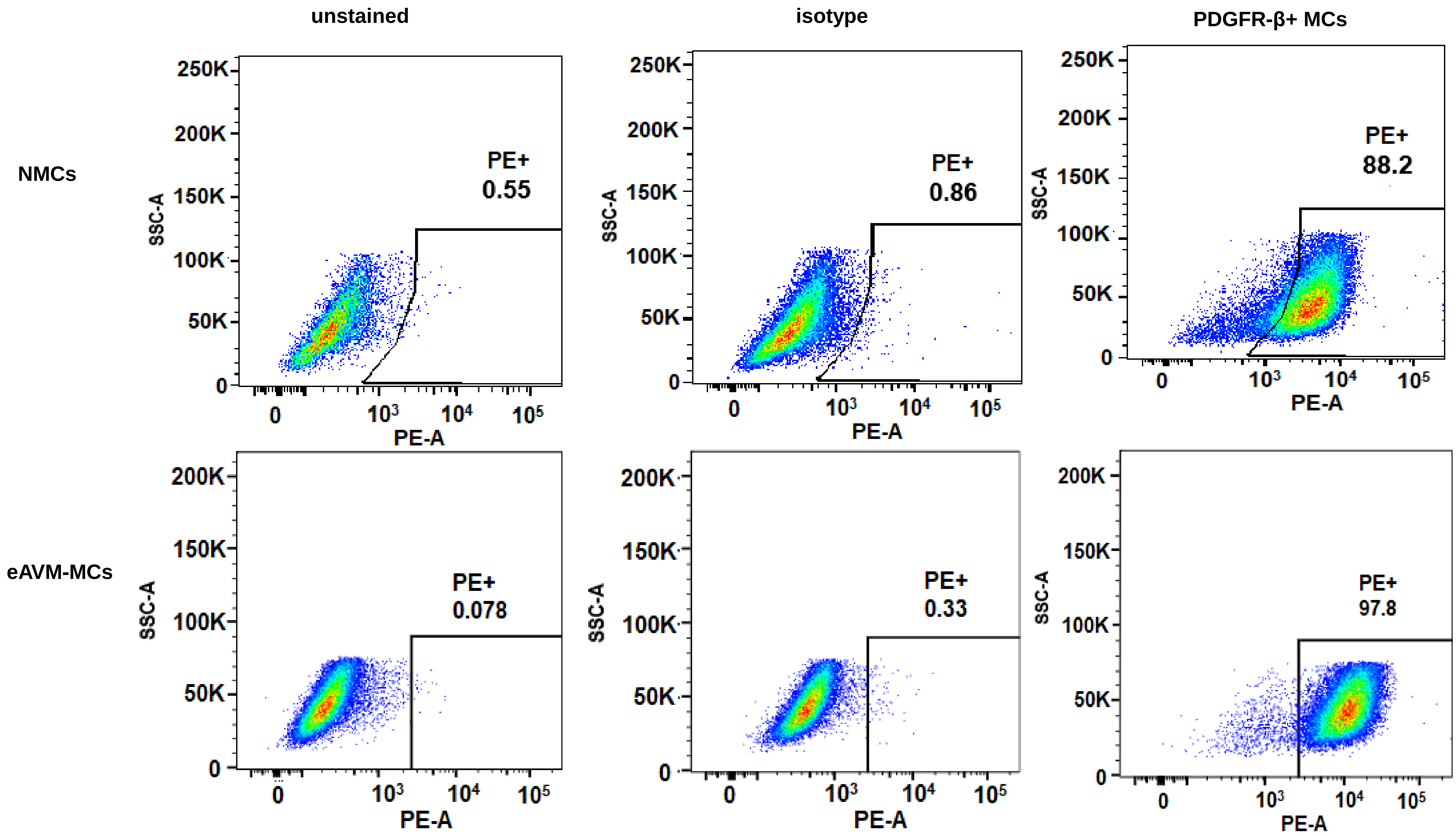

Figure S2. Flow cytometric analysis of mural cells isolated from eAVM and paired normal specimens. Fluorescence-activated cell sorting images demonstrate PDGFR-β+ MCs in isolated mural cells from eAVM and paired normal specimens.

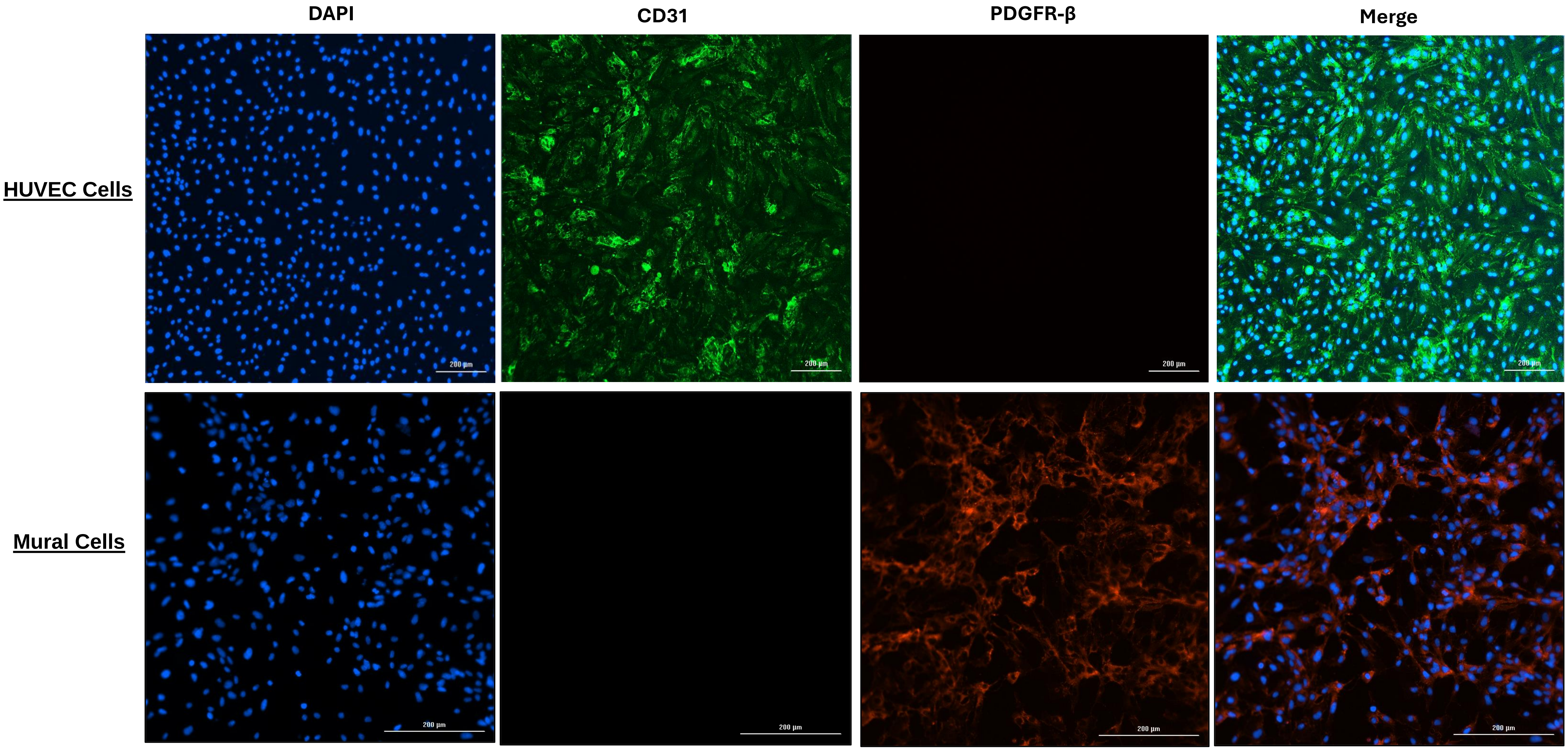

Figure S3. Representative image shows expression of CD31 and PDGFR-β in HUVEC and isolated mural cells before co-culture. Magnification =10x; Bar = 200 µm.

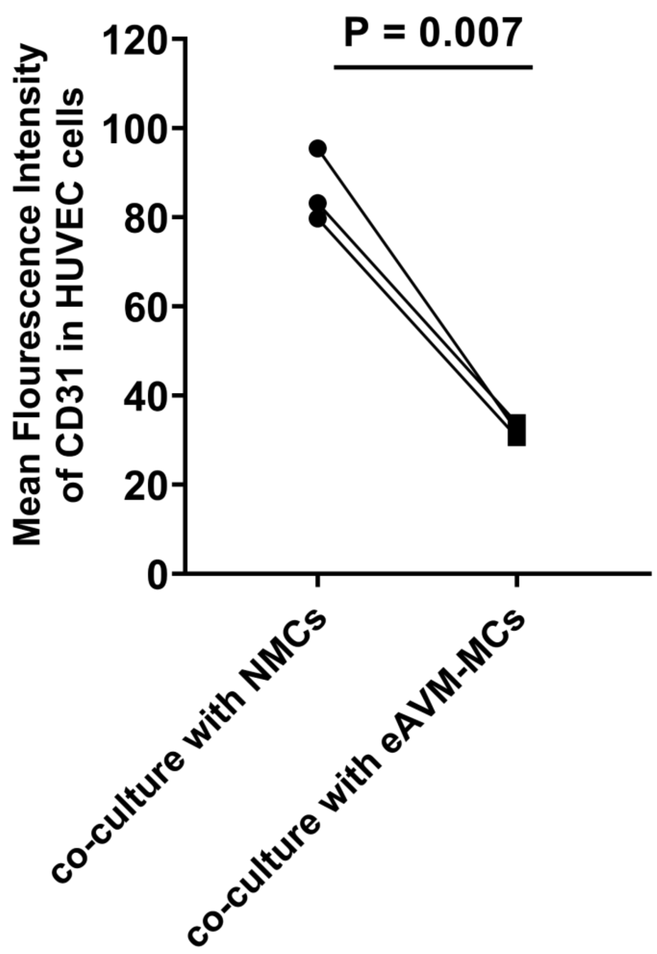

Figure S4: Fluorescence intensity of CD31 in HUVEC cells co-cultured with NMC and eAVM-MCs: The quantification of mean fluorescence intensity was analyzed by ImageJ. Data are expressed as means ± SD of 3 independent experiments. Paired two-tailed t-tests were performed to determine statistical significance ($P < 0.05$ was considered significant).

CD31 HUVEC

CD31 HUVEC

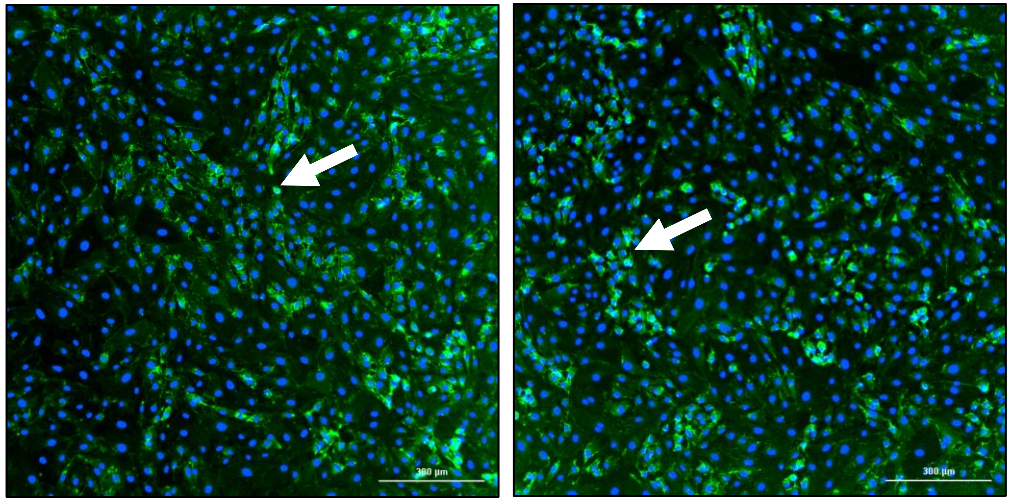

Before co-culture **After co-culture**

Figure S5. Indirect contact of eAVM's MCs does not affect CD31 expression in HUVEC cells. Representative image shows that CD31 expression is not reduced in HUVEC cells when co-cultured with eAVM MCs in an indirect manner. White arrows indicate CD31 expression. White arrows indicate the same cellular location. Magnification =20x; Bar = 300 µm.
