# Supplementary material for: Mural Cells Initiate Endothelial-to-Mesenchymal Transition in Adjacent Endothelial Cells in Extracranial AVMs
Source: Cells. 2024 Dec 21;13(24):2122. doi: 10.3390/cells13242122 (PMC11727354; doi:10.3390/cells13242122)
Supplement: Supplementary file 1 [file cells-13-02122-s001.zip › Revised Table S1.pdf]

Table S1. Antibodies used for Immunohistochemistry, flow cytometry and immunofluorescence.

Protein name	Application	Usage	Cat no.	Company
CD31	IHC, IF	1:800 (IHC), 1:800 (IF)	3528s	Cell Signaling
CD34	IHC	I:2000 (IHC)	PA5-85917	Invitrogen
PDGFR-β	IHC, IF	1:25 (IHC), 1:10 (IF)	LS-C99116	LS-BIO
PDGFR-β	Flow	20 µL/1x10^6 cells	MA1-10102	Invitrogen
N-cadherin (CDH2)	IHC	1:100 (IHC)	PA5-29570	Invitrogen
CD13	IF	1:50 (IF)	66211-1-1g	Proteintech
VE-Cadherin (CDH5)	IHC	1:20	36-1900	Invitrogen
SNAI1	IHC	1:50	PA5-115940	Invitrogen
