# Supplementary material for: Mural Cells Initiate Endothelial-to-Mesenchymal Transition in Adjacent Endothelial Cells in Extracranial AVMs
Source: Cells. 2024 Dec 21;13(24):2122. doi: 10.3390/cells13242122 (PMC11727354; doi:10.3390/cells13242122)
Supplement: Supplementary file 1 [file cells-13-02122-s001.zip › Revised Table S2.pdf]

Table S2. Primer/Probe for mRNA Assay.

Gene Symbol	Gene Name	Assay ID
ACTA2	actin, alpha 2, smooth muscle, aorta	Hs00426835_g1
CD31	platelet and endothelial cell adhesion molecule 1	Hs01065279_m1
CD34	CD34 molecule	Hs02576480_m1
CDH2	cadherin 2	Hs00983056_m1
SNAI1	snail family transcriptional repressor 1	Hs00195591_m1
SNAI2	snail family transcriptional repressor 2	Hs00161904_m1
VIM	Vimentin	Hs00958111_m1
CDH5	VE-Cadherin	Hs00901465_m1
